# An unexpected ferromagnetic foreign body detected during emergency magnetic resonance imaging: a case report

**DOI:** 10.1186/1756-0500-7-808

**Published:** 2014-11-18

**Authors:** Thomas Metterlein, Frank Haubner, Birgit Knoppke, Bernhard Graf, York Zausig

**Affiliations:** Department of Anesthesiology, University Hospital Regensburg, Franz-Josef-Strauss-Allee 11, 93051 Regensburg, Germany; Department of Otorhinolaryngology, University Hospital Regensburg, Regensburg, Germany; Department of Paediatrics and Juvenile Medicine, University Hospital Regensburg, Regensburg, Germany

**Keywords:** Metallic foreign body, Magnetic resonance imaging, Anesthesia

## Abstract

**Background:**

Sedation or anesthesia is often necessary in pediatrics when magnetic resonance imaging is performed. This anesthesia outside of the operation room combines specific requirements and risks. Ferromagnetic foreign bodies are a clear contraindication for magnetic resonance imaging due to the high magnetic field within the scanner. However, insertion of various small objects in mouth, nose or external auditory is not uncommon in small children and often remains unnoticed until specific symptoms develop. Early warning sings like movement of the object or heat development are then concealed by sedation or anesthesia preventing a timely termination of the possibly hazardous procedure.

**Case presentation:**

We present a case of a three year old Caucasian with an acute sinusitis due to unknown ferromagnetic foreign body in his nasal cavity. As soon as the suspicion was raised the procedure was aborted and the object that revealed to be a small button battery was successfully removed.

**Conclusions:**

The potential of unwelcome side effects and effective safety strategies of magnetic resonance imaging are discussed as well as the complications arising from ingested batteries.

## Background

There is a considerable growth in the number and types of procedures performed outside of the operation room. Many of them require sedation or anesthesia especially in diagnostic or interventional procedures in pediatric patients. Outside of the operation room anesthesia is characterized by specific requirements and risks. Furthermore, the anesthesiologist is faced with considerable pitfalls which do not exist in the operation room.

Magnetic resonance imaging (MRI) is a powerful non-invasive, radiation free diagnostic tool. The frequency of MRI examination especially in children who require sedation or anesthesia has increased in the recent years [1]. Here, the anesthesiologist has to be aware that ferromagnetic foreign bodies are a clear contraindication for a MRI scan due to potential relevant side effects [2]. However, accidental insertion or ingestion of foreign bodies often occur unnoticed in smaller children [3]. Symptoms develop with a certain latency initiating diagnostic workup possibly including MRI. Early warning signs indicating a ferromagnetic foreign body like movement of the object or heat development might be concealed due to general anesthesia preventing timely abortion of the procedure.

We report a case of a patient who underwent magnetic resonance imaging due to an acute sinusitis presenting with an unknown ferromagnetic foreign body in his nasal cavity as cause of infection.

Written informed consent was obtained from the patient’s parents for publication of this Case Report and any accompanying images. A copy of the written consent is available for review by the Editor-in-Chief of this journal.

## Case presentation

A nearly three year old male Caucasian (weight 14 kg) was admitted to the pediatric department of our hospital. The parents reported symptoms of an upper respiratory tract infection that had lasted for about ten days. During the last five days the boy developed a productive cough with fever. On the day of admission the parents recognized a diffuse swelling and redness of the forehead and the right orbital region.

An ongoing acute sinusitis was suspected and the child was admitted to the hospital. The preliminary physical examination revealed no further symptoms. A detailed inspection of nose and ears was not possible due to massive agitation and defensive behavior of the child. Laboratory results showed signs of a bacterial infection. Consequently intravenous antibiotics were administered.

In order to plan a possible surgical intervention an urgent MRI scan of the head was indicated by our otolaryngology department. Due to the uncooperativeness of the child and because of the intended immediate operative procedure it was decided to perform the MRI scan under general anesthesia.

Prior to induction standard anesthesia monitoring including peripheral oxygen saturation, EKG and non-invasive blood pressure was established. Anesthesia was induced with propofol (5 mg/kg) and remifentanil (0.4 μg/kg/min). To facilitate orotracheal intubation mivacurium (0.2 mg/kg) was administered. After successful intubation anesthesia was maintained with sevoflurane (1.8 vol% end expiratory concentration) and continuous infusion of remifentanil (0.1 μg/kg/min).

**Figure 1:**
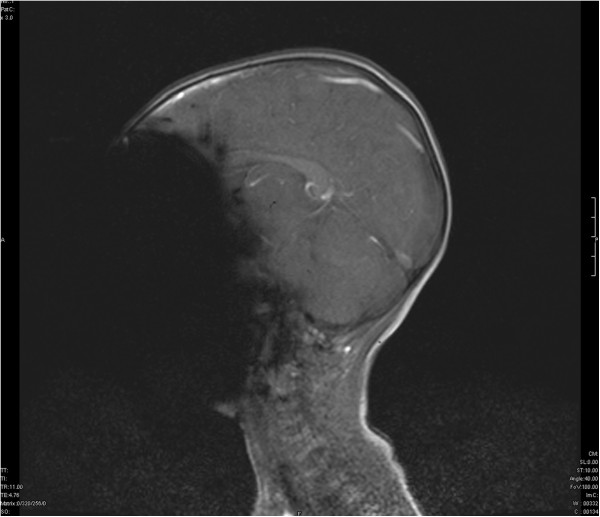
**Magnetic resonance image of the head** **(** **three year old child).** Elimination of the face because of a unknown ferromagnetic foreign body in the nasal cavity.

The child was positioned in the 3 Tesla MRI scanner and a preliminary scout was run. The first image revealed an extinction of the face indicating a ferromagnetic object in the scanned region (Figure 1). A quick inspection revealed no obvious foreign object in the facial region.

**Figure 2:**
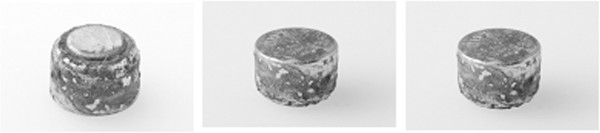
**Button battery removed from the right nostril of the three year old child.** After ten days in situ a relevant corrosion can be noted.

**Figure 3:**
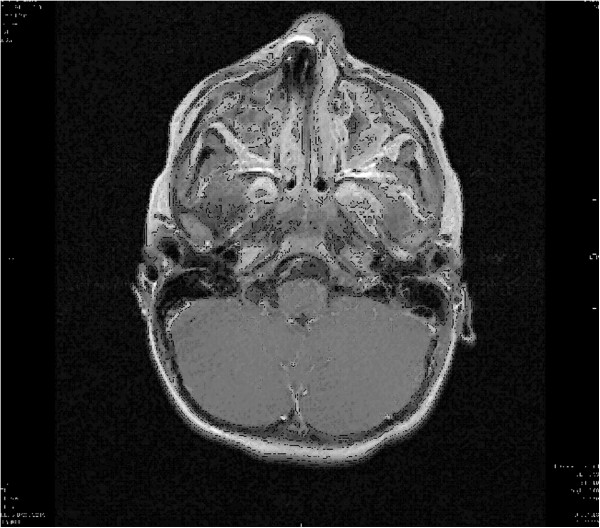
**Magnetic resonance image of the head** **(three year old child).** Elimination of the structures in the right nasal cavity because of remaining ferromagnetic material of the mucosa.

Therefore a ferromagnetic foreign body in either mouth or nose was suspected and the child was removed from the MRI scanner. The detailed inspection of the nasal cavity revealed a small foreign body that could easily be removed by colleagues from our ear nose throat (ENT) department. After complete removal the object was identified as a small button battery (Figure 2). A detailed examination revealed generalized swelling and a small mucosa lesion consistent with a burn or corrosion from acid. After a thorough lavage of the entire region the actual MRI scan was performed. While the initial scout was unremarkable detailed images still indicated ferromagnetic presence in the right nasal cavity (Figure 3).

To exclude any further foreign body a computed tomogram of the head was performed. The imaged proved that no further object was located in the nasal cavity. The structures seen on the MRI scans were considered as corresponding to ionic material in the mucosa.

After a second short inspection by the ENT colleagues anesthesia was discontinued and the child was extubated uneventfully.

The further post interventional course was unremarkable and the child was discharged from the hospital three days after the procedure. Oral antibiotics were continued for seven days.

On a follow-up visit four weeks later the child was doing remarkably well without any permanent issues.

## Discussion

MRI is a non-invasive, radiation-free diagnostic procedure [1]. A magnetic field with strength of 1.5 to 7 Tesla (T) (140 000 times the Earth’s magnetic field) is used to perform MRI clinically [3]. The frequency of MRI scans in children has increased in the recent years due to significant technical improvements opening a wide range of diagnostic possibilities without ionizing radiation [1]. Depending on the diagnostic needs the scan takes about 15 to 90 minutes. This procedure is extremely noisy and the patient has to be placed alone into a narrow pipe with limited access [2]. For optimum image quality the patients have to remain motionless. Younger children are mostly unable to cooperate adequately and therefore require sedation or even general anesthesia [1].

With increasing frequency of MRI for diagnostic purposes, anesthesiologists are more and more involved in this specific field.

The MRI environment in general has various peculiarities compared to the usual anesthesia environment and can to a certain degree be considered as “hostile” by anesthesiologists. Specific MR compatible equipment for monitoring, ventilation and drug application has to be used. Because of the limited space in the MRI scanner elongated tubing has to be used for ventilation or infusion. The increased dead space has to be considered, especially when small children are examined. During the actual scan the patient is left alone in the MRI scanner. Continuous clinical assessment (inspection, auscultation etc.) is not possible during the procedure and it has to be relied on monitoring systems. Emergency management is more difficult in the MRI environment. Access to the patient is extremely limited. Standard equipment (laryngoscope, defibrillator etc.) cannot be used. Transfer of the patient in a suitable area is time consuming and poses further risks like dislocation of the tube or loss of intravenous access. It also takes minutes after an emergency shutoff for the magnetic field to effectively decrease and furthermore leads to relevant expenses.

Because of the strong magnetic field no ferromagnetic materials can be used in the MRI scanner [2]. Metallic equipment is accelerated in the magnetic field [1]. Extreme caution has to be applied not to enter the scanner with any loose ferromagnetic material. Metallic foreign bodies present a specific hazard to the patient. They can be moved or twisted by the strong magnetic fields encountered in MR imaging studies. The seriousness of the risk depends on the ferromagnetic characteristics of the foreign body, its location, and the strength of the MR magnetic fields [2]. Fatal outcomes in patients with intracranial metal clips are described [4]. Another complication associated with ferromagnetic objects is local warming. The energy impulse during the scan results in a relevant heat development. The achieved temperature again depends on size, shape and ferromagnetic properties of the object [2]. Complications up to third degree burns are described [5]. Further complications can arise from the object itself. A relevant temperature increase of a battery results in a leak of the alkaline material.

In addition to the mentioned hazards, metallic material also produces relevant artifacts in MRI images [5]. The small button battery in the patient’s right nostril resulted in an almost complete elimination of the face (Figure 1). This results from a magnetic field inhomogeneity causing local signal elimination. Magnetic objects create their own magnetic field and dramatically alter behavior of protons nearby. Tissues adjacent to ferromagnetic components become influenced by the induced magnetic field of the metal object rather than the MRIs magnetic field and, therefore, fail to generate useful signals [5].

Microscopic pieces of metal deposited in skin or mucosa (tattoos etc.) are often invisible on radiographs, but they will produce visible MR imaging artifacts that are usually minor, although they can impair the diagnostic utility of a study [1]. This phenomenon was seen in the second MRI image taken (Figure 3). The right nasal cavity could not be assessed due to the artifact. Even after removal of the battery microscopic ferromagnetic particles seem to remain in the nasal mucosa.

Early symptoms of an undetected ferromagnetic foreign body in an MRI scanner would include movement of the object or minimal warming. Awake patients can describe a corresponding sensation at the location of the foreign body. In sedated or anesthetized patients these early warning signs are absent. All patients should therefore be thoroughly screened for foreign bodies before undergoing a magnetic resonance imaging study. Especially in pediatric patients a detailed physical examination might only be possible under sedation or general anesthesia. All involved physicians should be aware of this problem. A detailed examination for possible foreign bodies has to be performed or repeated prior to entering the MRI scanner.

Foreign objects in the aero-digestive tract are a serious problem in small children [6]. In the age group between two and four years the nose is the primary site of impacted foreign bodies [3]. Children in this age group are interested in their surrounding, move around and have acquired good skills in manipulating their fingers. Small toys are typical nasal foreign bodies while over the recent years the number of inserted button batteries has been increasing [6]. In general nasal foreign bodies can safely be removed compared to other sites (trachea, esophagus etc.). Pain and discomfort are the most common symptoms of nasal foreign bodies. Specific signs are unilateral foul-smelling nasal discharge [3]. Only a small percentage of the patients will present with epistaxis, but this is usually associated with failed attempts to remove the foreign body before presentation. Complications arise from unknown foreign bodies that cause complications from local infections to relevant tissue necrosis.

Miniature button batteries are common household items, powering many electronic devices and toys. Despite improvement in safety design of the products, children are still able to remove these batteries from the devices. Being small, they can easily be inserted into various orifices such as the nose, the ears and mouth. Inside the body cavity surrounding moisture results in corrosion of the battery casing. This can liberate its alkaline content (Figure 2). More important, the batteries can also generate local currents resulting in thermal burn and production of more alkaline materials through electrolysis. This results in extensive damage of the surrounding mucosa [7].

Even durations as short as 7 hours in the nasal cavity have resulted in septal perforations. An inserted or ingested battery has to be considered as medical emergency [3]. All types of batteries have to be removed as soon as possible. The risks of a compromised fasting period have to be discussed with all involved physicians and parents. They should be weight against the risk of potential long-term complications of battery injury.

## Conclusions

Performing an MRI with ferromagnetic foreign bodies poses various risks to the patient. Early warning signs might be disguised due to sedation or general anesthesia especially in infants and small children. It is therefore critical to eliminate the risk of an accidental exposure. A detailed physical examination if needed under sedation or general anesthesia has to performed prior to the MRI. It has to be defined who is responsible for this examination if different disciplines are involved.

Batteries are considered especially hazardous and therefore have to be removed as soon as possible.

### Consent

Written informed consent was obtained from the patient’s parents for publication of this case report and any accompanying images. A copy of the written consent is available for review by the Editor of this journal.

## Abbreviations

ENT: Ear nose throat
MRI: Magnetic resonance imaging
T: Tesla.

## References

[1] Paczynski S, Braun KP, Muller-Forell W (2007). Werner C [Pitfalls in magnetic resonance imaging. What should the anaesthesiologist know?]. Anaesthesist.

[2] Schenck JF (2000). Safety of strong, static magnetic fields. J Magn Reson Imaging.

[3] Kiger JR, Brenkert TE, Losek JD (2008). Nasal foreign body removal in children. Pediatr Emerg Care.

[4] Klucznik RP, Carrier DA, Pyka R, Haid RW (1993). Placement of a ferromagnetic intracerebral aneurysm clip in a magnetic field with a fatal outcome. Radiology.

[5] Hunter TB, Taljanovic MS (2003). Foreign bodies. Radiographics.

[6] Higo R, Matsumoto Y, Ichimura K, Kaga K (2003). Foreign bodies in the aerodigestive tract in pediatric patients. Auris Nasus Larynx.

[7] Loh WS, Leong JL, Tan HK (2003). Hazardous foreign bodies: complications and management of button batteries in nose. Ann Otol Rhinol Laryngol.

